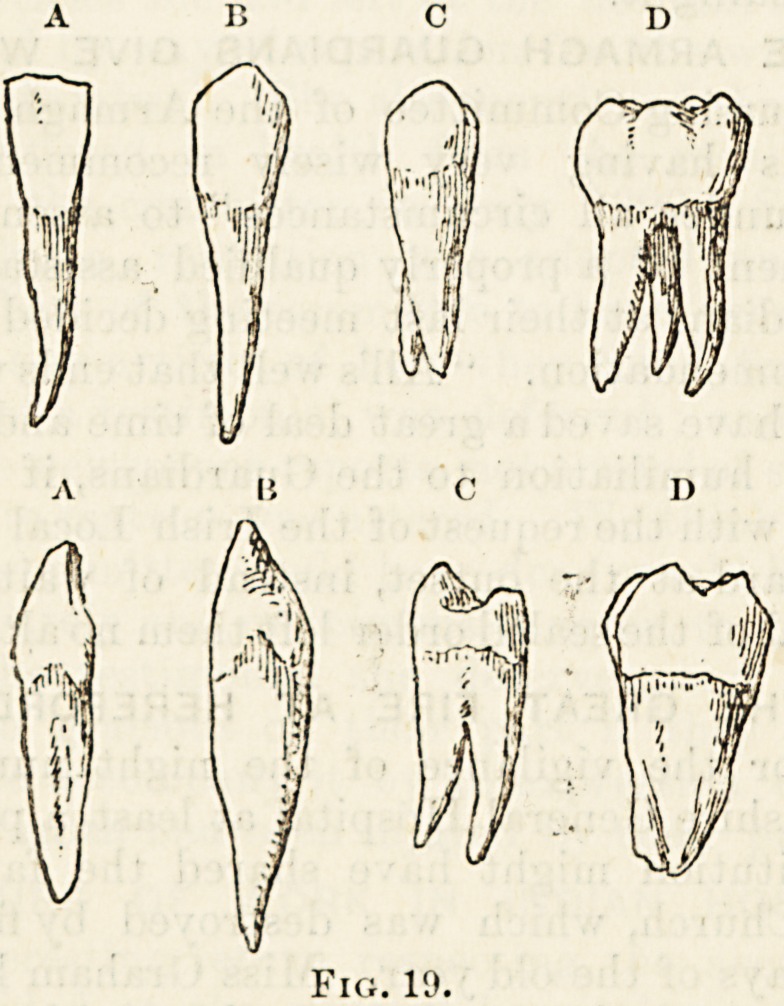# The Hospital. Nursing Section

**Published:** 1902-01-11

**Authors:** 


### The Hospital.

### Nursing Section.

Contributions for this Section of "The Hospital" should be addressed to the Editor, "The Hospital" Nursing Section, 28 & 29 Southampton Street, Strand, London, W.C.

No. 798.—Vol. XXXI. *SATURDAY, JANUARY 11, 1902.*

### Notes on News from the Nursing World.

### THE APPOINTMENT OF MATRON-IN-CHIEF OF THE ARMY SERVICE.

The impatient people who are clamouring for the announcement of the appointment of the Matron-in-Chief of Queen Alexandra's Imperial Military Nursing Service, must possess their souls in patience just a little longer. Colonel Sir Edward Ward, who has returned to his duties at the War Office, after a brief but much-needed rest, is fully aware of the importance of the choice, and of the significance which will be attached to it, and the question is receiving the most careful consideration. As more than one name has been mentioned in connection with the appointment, it may be as well to state that all rumours of the kind are purely conjectural. No information has yet been given officially, for the very sufficient reason that there is none to give.

### THE "ENGAGED" PROBATIONER.

Some of the daily papers have taken upon themselves to announce that it is useless for "engaged" girls to apply to be admitted as probationers at the principal hospitals. The basis for this announcement is clearly a letter from "An 'Unengaged' Nurse" which appeared in our issue of December 21st, for, though without acknowledgment, several of the statements made in that letter are cited in explanation of the course alleged to have been decided upon. But our correspondent, who alone is responsible for her assertions, only mentioned three matrons of "leading training schools" who had told her that they should not think of admitting a probationer if they knew her at the date of entrance to the hospital to be engaged. It is also implied that a general understanding has recently been arrived at between the doctors and matrons of our great charitable institutions that the "engaged" probationer shall be rigidly excluded. Of course matrons do not want probationers whose thoughts are intent on love affairs, but we doubt very much whether they are anything like unanimous in opinion that an "engaged" girl cannot under any circumstances discharge the duties of a probationer properly. At any rate, there has been no general promulgation of such a ukase as "No 'engaged' girls need apply," and we believe that a young woman who is able to prove her complete fitness as a candidate for training would not find her "engagement" an insuperable obstacle.

### THE WAR NURSES.

The *Bavarian*, which arrived at Southampton on Sunday morning, had on board, among the invalids, Nursing Sisters E. M. Beck and M. E. Howell. There were also on duty on the vessel Nursing Sisters M. H. Bruce, M. Whiteman, G. Napier, M. Rorke, and A. Kuys. The *Dunera*, which left the Cape for England on December 20th, has Nursing Sisters M. Stephenson and C. M. Mello on board as invalids, and for duty on voyage Nursing Sisters M. G. Hill' J. Creighton, M. Herring, M. Mavins, M. Meade' and W. D. Redstone.

### A SPLENDID EXAMPLE.

Two facts in connection with the building of the new Nurses' Quarters of the Victoria Caste and Gosha Hospital, Madras, rendered the opening ceremony, which took place towards the end of last year, especially interesting. First, the Government Architect, Mr. G. S. T. Harris, had drawn up the plans of the home, and supervised the building entirely free of charge, and the contractor had so substantially reduced his charges that the whole expenditure is not expected to exceed Rs.4,000. Everything is of the best quality. The structure is of red brick and timber with stone facings, and contains a central sitting-room and the necessary accommodation for five nurses. The home has been paid for and presented to the hospital by Mr. Krishnadoss Balamukandoss, who, reading in the Visitors' Book that Lady Ampthill thought that new nurses' quarters were urgently needed, at once came forward and promised to give all the money required. His munificence has benefited the hospital directly as well as indirectly, for the old nurses' quarters can now be used for isolating septic cases which in the interest of the other patients the authorities had hitherto been obliged to refuse.

### SCOTLAND AND THE COLONIAL NURSING ASSOCIATION.

The appeal issued by the Lord Provost of Glasgow in connection with the work of the Scottish branch of the Colonial Nursing Association, following the public meeting last month, has met so far with a liberal response. There is no doubt that a marked impression was created by the excellent speeches delivered at the meeting. Moreover, the memoranda prepared by the ladies who are moving in the matter, which is sent to everyone who is invited to contribute, puts the objects of the Association in a trenchant manner. We hope that here, as well as on the other side of the Border, it will receive in the present year the large measure of support which it so well deserves from all classes.

### DISTRICT NURSING IN HONG KONG.

The first annual meeting of the Hong Kong Nursing Association was held at the end of November. It was inaugurated in the early part of the year, but the particulars placed before the committee only included the experience of three months, because it seemed desirable that the meeting should take place in cool weather. During these three months—which terminated on September 30th—the two nurses had been employed almost all the time, and the chairman said that the institution "had proved a great success and filled a great want in the colony." There had been a slight deficit on the working at the time the report was written but that had since been made up, and the guarantee fund now stood again at the original sum. Two points were raised, both of much importance. During September, October, and November two other nurses —one from Shanghai and another a resident in the colony—had assisted the two institution nurses, or, owing to the number of cases, they would have been in serious difficulties. So that practically four nurses had been at work. Therefore the question of permanently employing another nurse would soon have to be decided. The other matter was the housing of the nurses. Hitherto they have been resident at the Peak Hospital, and the arrangement has been found to work well, as the position is very central, and the nurses appreciate having the companionship of other nurses off duty. With regard to the erection of a permanent nurses' home, nothing can be done until the requisite funds are forthcoming, and even when funds are available it will not be an easy matter to find a suitable site. But assuming that the Colonial Office do not object to the continuance of the existing arrangement in the interval, it seems admirably to meet the case.

### THE NEW MATRON OF SOUTHAMPTON WORKHOUSE INFIRMARY.

We congratulate the Southampton Board of Guardians, the patients of the workhouse infirmary, and the nursing staff on the excellent appointment which has been made to the post of matron. Miss Ballantyne commenced her nursing career as probationer at the Cottage Hospital, High Barnet, after which she received three years' training at Guy's Hospital. In September, 1897, she became sister at Lewisham Infirmary, and recently she has discharged the duties of assistant matron. Miss Ballantyne belongs emphatically to the class from which the matrons of important workhouse hospitals should be selected, and the choice augurs well for the future of Southampton Infirmary.

### NOVELTIES IN DECORATIONS.

It becomes increasingly difficult each Christmas for the sisters and nurses of hospitals and infirmaries to impart originality to their schemes of decoration, but several novel features seem to have been introduced this year. Instead of simply hanging up flags, Chinese lanterns, and wreaths of evergreen, a successful attempt has been made in some quarters to give a special feature to each ward, with the idea of making it quite distinct from its fellows. At the Brentford Union Infirmary, Isleworth, one of the wards was transformed in part into a pond, upon whose glassy surface a group of white wool ducks gaily disported themselves. In another a centre table had become for the nonce a rocky coast, from which rose a lighthouse, the work of the night nurse in the few hours at her disposal for the making of masonry. At the Chelsea Hospital for Women the honours on one floor were divided between the heroes and heroines of nursery rhymes and the characteristics of distant lands: Bo-Peep, Red Riding Hood, and Hensel and Gretel, each with suitable surroundings, appeared on different tables, whilst some of the salient features of Japan and India had been borrowed for the occasion for the larger wards, and even Lapland with its snow and ice and its diminutive Lapps occupied the end of one of the passages. The Coronation ceremonies of the coming year will probably suggest decorations to wideawake nurses for next Christmas.

### THE BELPER LIBEL CASE.

The committee of the Belper Isolation Hospital cannot be congratulated upon the manner in which they have so far treated the libel suit, resulting in the complete triumph of the late matron. After a very curious discussion respecting a strange proposal that Dr. Allen should receive pecuniary assistance in order to lighten the burden of the damages awarded to Miss Martin, a letter from the late matron was considered. Miss Martin asked for the return of certain articles she had left at the hospital and for a testimonial by way of reparation for the wrong done to her in dismissal. She also mentioned that she had greatly suffered in health from the result of the action of the committee in July 1900. Somewhat grudgingly, a resolution was adopted authorising two members of the committee to hand over to Miss Martin "any articles of hers they could find"; the question of a testimonial was deferred; and, according to the newspaper reports, not a word of sympathy for the late matron was uttered. We think that the least the committee could have done would have been to immediately and unanimously agree to give Miss Martin the testimonial she deserves, and it would have been gracious on their part if they had also decided to accompany it by an expression of regret that her health had been injured by their procedure.

### THE HOURS OF WORK IN INDIAN HOSPITALS.

An interesting return respecting the accommodation provided at the general hospitals at Calcutta, Bombay, Madras, and Rangoon, has been issued by the Superintendent at Rangoon, but there is no information respecting the hours of duty, and, except in connection with the Madras hospital, the name of nurse is not mentioned. This is a matter for regret, because there is a strong opinion that the working hours of nurses in the Presidency towns are unduly long. In fact, at Rangoon we learn that nurses are constantly breaking down under the strain of the excessive hours, and particularly under that of three months' consecutive night work. The silence of the Superintendent of the Rangoon hospital on the question has naturally confirmed, rather than dissipated, the idea that the nurses are overworked, though the matron states from her own experience that three months' consecutive night work, from 6 P.M. to 6 A.M. is a better arrangement than one month. Her argument is that after the first week a nurse gets accustomed to the routine of night hours and night meals, and that it is preferable to have a first week once in six months than once in two. As we lately stated, the nursing staff at Rangoon has been considerably augmented since the hospital was taken over by the municipality, but the chief reason given against a further augmentation is the absence of room. In that case, it is the clear duty of the municipality to provide the accommodation needed.

### MARRIAGE NO IMPEDIMENT.

In the Buxton "Parish Magazine" there are some interesting details of the marriage of Miss Marian Sheridan, Queen's Nurse, of Buxton, to the Rev. J. C. Holden, one of the assistant clergy of the parish The wedding took place at the parish church, Seaham Harbour, Durham, early in the morning, and was followed by a celebration of Holy Communion. Among the presents received by the bride was a valuable and beautiful travelling clock, presented by the committee of the Buxton District Nursing Association. The present was accompanied by a letter expressing, through the president, the great satisfaction of the committee that Mrs. Holden feels able to continue her "much appreciated work among the sick and poor of Buxton." The decision of Mrs. Holden is an evidence of the fact that marriage does not necessarily preclude a nurse who is devoted to her work from pursuing it.

### THE ARMAGH GUARDIANS GIVE WAY.

The Nursing Committee of the Armagh Board of Guardians having very wisely recommended the Board "under all circumstances" to assent to the appointment of a properly qualified assistant nurse, the Guardians at their last meeting decided to adopt the recommendation. "All's well that ends well," but it would have saved a great deal of time and trouble, and some humiliation to the Guardians, if they had complied with the request of the Irish Local Government Board at the outset, instead of waiting until the arrival of the sealed order left them no alternative.

### THE GREAT FIRE AT HEREFORD.

But for the vigilance of the night nurse at the Herefordshire General Hospital at least a portion of that institution might have shared the fate of St. James's Church, which was destroyed by fire in the closing days of the old year. Miss Graham happened to look out of the window at three o'clock in the morning, and saw what appeared to be a tongue of flame issuing from the extreme east end of the church, which adjoined some of the out-buildings of the hospital. She at once asked Miss Dew, who was also on duty, to telephone to the National Exchange Office in East Street a message for transmission to the city police station that St. James's Church was on fire, and though the conflagration could not be got under until the church was destroyed, her action showed the presence of mind which is so much needed in the nursing vocation. Miss Graham also very properly roused Miss Elphick, the matron, and subsequently the whole of the nursing staff watched the progress of the fire, having been previously assured that there was no danger of it spreading to any building in the vicinity. Both themselves and the patients were, however, somewhat alarmed at the appalling blaze—so appalling that there was a red glare upon the patches of ice and snow on roof and roads and meadow land, and they could see the woods and hills across the river as clearly as if it had been daylight—especially as now and again they actually felt the wave of heat come across from the church with sufficient intensity to make the panes of glass in the hospital windows hot.

### TONBRIDGE UNION INFIRMARY.

After two courses of lectures on Anatomy and Surgical Nursing, and Physiology and Medical Nursing, "given by the medical officer to the probationers of Tonbridge Union Infirmary," examinations were held, Nurse Morgan obtaining the highest number of marks in the latter subject, and Nurse Garrett in the former. The matron had promised a prize for the best result of a year's work, but both nurses having done equally well it had to be divided, each nurse receiving a very useful nursing book. The prize is to be presented annually.

### PROGRESS AT NEWCASTLE-ON-TYNE.

An improvement of considerable importance has been effected at Newcastle-on-Tyne Workhouse Hospital. The inmates are no longer allowed to assist in the nursing of the sick, an adequate staff of fully-trained nurses having at length been provided. This is as it should be in a city of such size and wealth as the metropolis of the North. By degrees the untrained matrons of workhouse infirmaries will go the way of the pauper attendants. But the time it has taken to bring about the reform at Newcastle shows that there must be no relaxation of effort.

### COMPETITION FOR A BLACKPOOL POST.

It is satisfactory to learn that there is no difficulty in securing the services of a thoroughly competent person for the position of matron of an infectious diseases hospital, if the conditions of the appointment are reasonable. A very experienced nurse has just been appointed as head of the nursing staff at Blackpool Infectious Diseases Hospital, and the Sanitary Committee of the Blackpool Town Council had no less than 65 applications to consider. The appointment is, of course, subject to confirmation by the council, but this in view of Miss Florence Brown's excellent credentials is not likely to be refused. Perhaps the attractions of the famous Lancashire watering place had a little to do with the number of applications.

### AN OVERDOSE OF SULPHONAL.

Sulphonal being considered such a safe narcotic, overdoses are rare, and the following case may therefore be interesting to nurses. According to a ccorresdondent an elderly lady, who had been in the habit of taking sulphonal for sleeplessness, took the fatal dose, it is thought, about 9 a.m. one morning. The maid gave her tea at 8 a.m., and said she talked and appeared as if nothing unusual had happened. In about an hour the maid found her in a heavy state, and although she spoke she soon lapsed into unconsciousness; so the doctor was called in and a nurse was engaged the same evening. The nurse says that to all appearance the patient was sleeping quite naturally, eyes shut but pupils dilated, and continues, "if lying on her back she snored, but when put on her side was perfectly quiet; but she never stirred herself or moved a hand even, and only responded to the battery if it was placed at the corner of her mouth, then the response was merley a slight twitch. She slept on without any apparent change for 60 hours, and then passed quietly away in her sleep. The temperature, which was normal at first, gradually rose, and was 105° at death. The pulse kept strong until almost the end, and never went higher than 108, and the first 48 hours was between 80 and 90. The treatment ordered was hypodermic injections of strychnine, enema injections of salt and bicarbonate of soda, transfusions of salt and water. The nourishment was egg, milk and brandy and beef essence, which was given by the mouth and the rectum. The dose supposed to have been taken was 180 grs., as the empty box was found and it had just been filled."

### Lectures to Nurses on Anatomy.

By W. JOHNSON SMITH, F.R.C.S., Principal Medical Officer, Seamen's Hospital, Greenwich.

### LECTURE VIII.—THE FŒTAL SKULL.—THE TEETH.

*The Fœtal Skull.*—The skull of the new-born child, especially the upper part, differs in many respects from that of the adult. The cranial or upper part is much larger in proportion to the whole skeleton, whilst the bones of the face are much smaller. The top and sides of the cranium do not present an even and unyielding surface of hard bone, but are composed partly of bone and partly of fibrous membrane. The different cranial bones described in the last two lectures can be distinctly made out, but they are not yet joined together, and can be moved on one another and even made to overlap. These bones are thin and soft, and in the case of injury to the head are more liable to be bent and depressed than to be fractured. A fall, or blow, or a squeeze of the head likely to result in a "grown-up" subject in a severe depressed fracture of the vault of the cranium would probably cause in the infant a deep and cup-shaped depression of bone, which in the course of a few weeks would probably right itself in consequence of the elasticity of the injured bone. The frontal bone in the fœtal skull consists of two lateral halves divided by a suture extending to the root of the nose. The upper expanded part of the occipital bone is detached from the lower or basal portion which surrounds the foramen magnum.

*The Teeth.*—Although, as has already been stated, teeth are not bones, we shall find it convenient to describe these structures in connection with the skull, and not, as is done in most anatomical works, with the organs of digestion.

In man the teeth being used simply for dividing food are small, even, and regularly arranged, and differ very much in form and size from the teeth and tusks of many animals who use these organs also for attack and defence, for prehension, for digging, and even as aids to locomotion.

The architecture of the human tooth is simple, and undergoes but slight modification. There are the exposed portion, the *crown* as it is called (fig. 18, A), with its surface of hard and white enamel, and the imbedded portion, the *root* or fang (B) in which the enamel is replaced by a substance

FIG. 18.

resembling in appearance and also in minute structure true bone; this substance is called *cement*. The main portion of the tooth is made up of a hard tissue (*dentine*) (C) composed partly of animal partly of earthy matter. Enclosed by the dentine is a distinct cavity (*the pulp cavity*) the shape of which corresponds very closely to that of the tooth. The slight construction between the crown and the root is called the *neck* of the tooth (D).

The second and final dental equipment which from the seventh to the eighteenth year of life has been gradually substituted for the so-called *milk* or temporary teeth of childhood, consists, when completed, of 32 teeth arranged symmetrically in both jaws and on both sides. Thus there are, or should be, in each jaw 16 teeth, eight on one side of the face, eight on the other.

The four central teeth in each row are called *incisors*, the single larger tooth on each side of these is the *canine*, then we come on the *premolars* two on each side of the canines, and finally to the three grinding teeth or *molars*.

It is usual in anatomical works to express the number and arrangement of the teeth both in man and animals by what are called dental formulæ. The teeth are indicated by the initial letters of their distinguishing names—i for incisors, c for canines—and the numbers above and below the line represent the teeth on *one side* of each jaw. The following is the dental formula of the second or so-called permanent

FIG. 19.

set in man. This, it should be borne in mind, represents only the teeth on one side.

$$i\frac{2}{2} \quad c\frac{1}{1} \quad p\frac{2}{2} \quad m\frac{3}{3} = 16.$$

The following is the dental formula of the first set which contains only 20 teeth.

$$i\frac{2}{2} \quad c\frac{1}{1} \quad m\frac{2}{2} = 10.$$

The *Incisors* (fig. 19A).—These are used for simply dividing or cutting food. The crown of each is rectangular in front, sometimes almost square but usually oblong, the length exceeding the breadth. The anterior surface which is slightly convex and the posterior surface which is concave, meet in a sharp cutting edge at the free margin of the tooth. The *undivided* root is long and straight, and a little compressed from side to side.

The incisors are smaller in the lower than those in the upper row, and in the latter row the two middle teeth are larger than those on either side.

The *Canines* (fig. 19B). These, which are the representatives in man of the well-marked and projecting teeth in the carnivora, and the tusks of the narwhal and walrus, have each a thicker crown and a longer root than the incisors. The upper margin does not present a broad cutting edge, but is angular, and terminates in a blunt point or cusp.

The *Premolars* or *Bicuspids* (fig. 19C). In each tooth the top of the crown is flattened and presents two blunted points or cusps. The root, which is shorter and flatter than that of a canine tooth, is often forked.

The *Molars* (fig. 19D) have each a large square crown with a broad upper surface, on which are set four or five cusps. In the upper row each molar has three diverging fangs—two in front, one behind; in the lower row each molar has only two roots placed side by side.

It was pointed out by Sir Charles Bell that a premolar is like two canines incorporated, and that a molar may be imagined as a tooth in which four canines are welded together.

The first appearance or "cutting" of the milk teeth follows a common but not invariable course. The rate of growth, and the size and even the form of these nascent structures are influenced to some extent by the general health of the child, and the conditions, both inherited and acquired affecting its development.

When the child is six months old the nurse begins to look for the middle incisors. These are followed in the course of the next six weeks or two months by the lateral incisors. At the end of the first year of life the child should be supplied with all its incisors and four molars, the first one on both sides and in each jaw. Next follow the canines about the middle of the second year, and by the end of the third year the first or milk set should be complete, the child presenting in each row four incisors, two canines, and four molars.

As these milk teeth become loose in consequence of the pressure of the new teeth and their fangs become absorbed, they are gradually shed and replaced by their successors in the following order:—

The first of the adult teeth to appear are the first molars. These, which are usually cut soon after the age of six years, are followed in the course of the next four years by the eruption first of the two middle incisors, next of the two lateral incisors, and, after these, of the bicuspids. The canines are late as they are not often seen until the eleventh year. In the "school" period of life the second molars make their appearance, and, finally, when the third decade of life is impending, the third molars or wisdom teeth are added.

### Sick-Room Cookery.

By Maud Mason.

The relation of dietetics to the proper treatment of an invalid has been so often and so persistently insisted upon by those who have studied the subject, that the importance of the matter is in itself sufficient justification for bringing before nurses the value and necessity of good sick-room cookery. Sir Henry Thompson has said that "No man is a really accomplished physician or surgeon who has not made dietetic principles and practice an important part of his professional education." It is surely, then, befitting that a nurse, particularly one in charge of private patients, should understand the different ways of preparing the foods contained in the limited dietary of an invalid, and should be acquainted with the various kinds suitable for the feeding of an invalid suffering from certain diseases. If she has grasped the great importance of this, no nurse will consider her training complete unless she has had opportunities given to her of studying practically this branch of her profession. It is gratifying to know that this is being realised, not only by those in hospitals who are responsible for the training of the nurses by allowing the members of their staff to attend lessons in sick-room cookery, but by the nurses themselves. Those who have gone out to nurse private cases soon find the disadvantage of the lack of this knowledge, and some have set about attending courses of sick-room cookery on their own account after their training, but properly speaking such knowledge should have been acquired before they were considered qualified nurses.

### The Importance of Knowledge.

Miss Nightingale has in no measured terms enforced the lesson that sick-room cookery should be understood by the nurse, and Dr. Pavy maintains that "it is not too much to say that success in the treatment of disease is largely dependent upon a display of judicious management with regard to food." One cannot help feeling surprised, when the matter has been dealt with so authoritatively, that there are still, comparatively speaking, so few nurses who have studied sick-room cookery, and so many who think it an uninteresting branch of their profession. And having gained this knowledge, of how much more value are the nurse's services, not only to the medical man, but also to those stretched on beds of pain? The doctor has drawn up, or suggested a dietary, when he sees his patient for a few minutes in the day, and it remains for the nurse, or probably some incompetent, though doubtless willing, housewife or cook to see that the patient has his milk, his beef tea, his little piece of fish, etc., at a proper time, but alas! not always in the proper way. These remarks, necessarily, apply especially to nursing in private houses where the nurse has a more responsible and individual position.

### Cleanliness.

At the outset let me offer from a practical standpoint a few suggestions to those who have to cook for an invalid. Above all things, remember that cleanliness is of paramount importance in all branches of cookery, and most of all in that for the sick-room. Carry with you into the kitchen the same sense of order and neatness which guides you in the hospital ward, or in the chamber of the sick; never allow yourself to merely depend on other people's eyes; use your own, and do not be afraid of being considered too particular; better err on that side than on the other.

### Punctuality.

With regard to the time at which the food is given, punctuality is another great virtue I should like to emphasise. No one knows better than a nurse how fickle is the appetite of an invalid, and no one is better pleased than she is when he begins to show signs of appreciation of his food, but woe betide all efforts at success if the promised or expected dainty is late in making its appearance! The desire which the nurse fostered has outlived itself and only disappointment follows. It is no good blaming the patient's appetite —the cook, whoever she is, like Mrs. Hawkins, has "only herself to blame."

### Variety.

Probably the most difficult thing to accomplish in an invalid's dietary is to provide variety. The restrictions under this heading are so great that it is indeed very often a hard nut to crack. The materials from which one may make a selection are so few that one has all the more need for a knowledge of the different ways in which food may be served. And here the nurse meets with the greatest encouragement, because the more often she tries her hand the richer does she become in ideas. But the seed must be sown in order to reap the hundredfold. The use of simple flavouring agents is a great help here, provided they are not forbidden. Lemon rind cut very thinly, without the white underpart, makes one of the most acceptable flavours for milk custards and sweet puddings; cinnamon, using the stick, not the powder, is also very acceptable for gruels, barley water, rice water, etc. Soups, which are often so horribly tasteless, may easily be made more palatable by boiling vegetables and herbs in them. Cut up the vegetables, such as carrot, turnip and onion, tie them with some herbs (parsley, thyme, etc.) in a piece of muslin, and after boiling them in the soup, express the juices, leaving the solid matter behind.

### The Individual Taste.

A word is called for with regard to the consulting of the individual taste. It is no use trying to force a food on a patient merely because it ought to be good for him, respect and value his likes and dislikes which are probably the surest signs as to whether the particular food will be digested by him. Try to get the food dished up hot. In case it has to be kept hot after it is cooked, it is a good plan to place it in a covered basin, and stand the basin in a pan of hot water, keeping the water gently simmering; the food will remain hot without deteriorating by being over-cooked.

The Tray.

The necessity and the pleasure of offering food to an invalid in a pleasing and attractive fashion leads to a consideration of the tray on which the food makes its appearance. Here, especially, avoid any appearance of slovenliness—it is well worth an effort to see that cloth and silver are spotless. A vase of flowers, even one flower or some prettily tinted leaves all play a charming part on the invalid's tray, if they are procurable, though strongly scented flowers should be avoided.

(*To be continued.*)

### Nursing in Pietersburg.

By an American Nurse.

Pietersburg is the largest town in the Transvaal north of Pretoria, and our last hospital on the Pietersburg line is stationed here. I obtained leave to run up to visit the town, although the railroad is still rather dangerous to travel on, and the trains few and far between, sometimes only one a week. We started with an armoured train and ration train, whilst our own consisted principally of trucks filled with goods, horses, and the train escort in armoured trucks. There were only two carriages, a compartment was cleaned out for myself and two officers, and with that we had to be content for two days, but no travelling was allowed at night. Some of the scenery we passed through was grand and wild, we also crossed several rivers, the bridges guarded by troops who are always glad to get the books and papers we throw out to them. We had a Post office on board and it was amusing to see the eagerness the men showed in crowding around the train. Some got letters which were handed out in packages; others bought stamps and postal orders so as to send money home for Christmas; one asked me for writing-paper, and I was glad to be able to give him some and a packet of envelopes. These camps are dreadfully lonely, the soldiers are stationed here to watch the line as there are not many blockhouses up so far, the only diversion they have is the passing of the trains, and that seldom happens. The country looked at its best, the veldt and trees quite green, many in blossom, and some of the veldt flowers are lovely.

The Town and Hospital.

We arrived in Pietersburg on November 9th, the King's birthday, in time to be present at the Gymkhana held in honour of that event. The convalescent patients attended, and the troops took part in the games with the officers, the Wilts, Gordons, and Bush Veldt Carbineers. There were the usual sports, polo included. The officers served tea during the interval, and it was much appreciated by the nursing sisters and a few guests. Pietersburg is on the high veldt, and is much more healthful than the places further south, but not nearly so pretty as Warmbaths. There are few kopjes, but high mountains can be seen in every direction. The Zand river runs through Pietersburg, an insignificant stream during the winter, but in the summer quite a torrent. The town is much like other Dutch towns, built around a square which serves as a market place in times of peace. The hospital is equipped for 100 beds; Major Wilson, R.A.M.C., in charge with Mr. Johnson, civil surgeon, and three sisters. Sister McGowan is the acting superintendent. The hospital and quarters, are in tents of the Indian pattern and very comfortable. There is not much enteric at present. The Major pays particular attention to the sanitary condition of the camps under his jurisdiction, and enforces the order to have all water boiled wherever he can.

The Kaffir Hospital.

I was much interested in the Kaffir Hospital, under the care of Dr. Johnson and Sister Lawson, who take on this duty voluntarily in addition to their other work. Were it not for the kindness of these Good Samaritans these poor creatures would be left to suffer until death relieved them, for no provision is made for the Kaffirs in these outside places. There is a Kaffir hospital in Pretoria, and perhaps other large towns, but I know of no other place where they are given treatment except Warmbaths. At Warmbaths a large out-patient practice is carried on, and many women and children, as well as men, with all sorts of diseases are treated. At Pietersburg, however, they are all surgical cases, including a few gun-shot wounds, from the sniping of the Boers. The doctor operated on a case of long standing just before my visit, and it was doing remarkably well. It is really marvellous how quickly the Kaffir flesh heals if it has only a little attention; I have done a little grafting myself, and in every case it has been a success.

The Refugee Camp.

Another interesting place to visit at Pietersburg is the refugee camp. There are nearly 5,000 refugees, men, women and children, living as families in a tented city. The camp looked as well as one could expect, clean but not tidy, How could it be tidy when in some bell-tents two grown people and nine children live all together? The floors of the tents are what they call "smeared"; some kind of composition is smeared on like mud, and when dry it is quite hard. Some of the families have little gardens around their tents, and others keep chickens and goats. I saw some burghers building a brick house, but I hope things will be settled before it is finished, and they will not need it. Anyhow, it gives employment to several men who are working on it. I also visited the hospital. Two of our reserve sisters are in charge, with several Dutch nurses under them instead of orderlies. There was one marquee for the children. Some of the convalescents were learning to speak English. The pale little faces told their own tale. Most of them had large blue eyes and tow-coloured hair, and they seemed pleased to be spoken to. I was surprised that they could answer so well in English; the women too seemed glad to be noticed, but could not speak as well as the children. Only the bad cases are taken into the hospital. During the epidemic of measles the mortality has been high—500 deaths since last May, the majority being among the children. But now that we have our own doctors and sisters in charge it will make a difference, and everything is in better working order than at first. There is a school for the children, and big lads attend; all the studies are in English, though I understand that the teachers can speak both languages. The lady commissioners arrived the night before I left Pietersburg.

Literary Crumbs.

On our way down we distributed more literature. The sisters enjoy throwing out the reading matter, which some kind friends collect for the block-houses on the line. The men begin to look for it now, and it does not need a second bidding when they see a sister at the train window with a bundle of papers. They begin to make tracks for the line at once. Some of the block-houses are quite a distance from the railroad, especially those on the kopjes, but perhaps the books and papers are passed on to them.

### The District Nurse in America.

A NEW DEPARTURE AT A BOSTON HOSPITAL.

IN America, as in England, the district nurse is undergoing the process of development, and her importance is being more and more recognised. At the graduation of nurses from the training school of the New England Baptist Hospital at Parker Hill Avenue, Roxbury, near Brookline, Boston, it was announced that the trustees of the institution felt that the time had come for it to address itself to a wider service in ministering to the sick in the homes of the poor. The hospital was only incorporated in September 1893, but it has done a constantly increasing amount of free work from its own earnings. Thus in 1900, 52 cases were nursed without charge, and 28 at half the usual rates. But it was felt that in the districts of the city of Boston which are reached by the Baptist mission churches there were still large numbers of sick people who could not be accommodated in the hospital, and could not conveniently be removed to it. As to accommodation, it is hoped that in the not distant future a new hospital building on a large scale, adequate to proved requirements, may be erected on the site which has been secured adjoining the present building. Meanwhile, it has been decided to inaugurate a system of district nursing which shall be maintained as a part of the discipline and education of the nurses in the training school, by which each nurse in her last year shall be required to give six months' service in district nursing.

THE QUESTION OF ACCOMMODATION.

To carry out this scheme, however, a nurses' home is indispensable. At present the accommodation provided for the nursing staff consists of seven bedrooms and a bathroom. As there are twelve nurses, most of them sleep two in a room. One room is given to the head nurse, and the other single room is so small that only one bed can be placed in it. What was once the reception room is now used as a bedroom. There is no room in the house devoted to social purposes. The nurses possess a piano, but as it has to stand in the small hallway, they cannot gather together after a hard day's work either for social chat or for music. The night nurses have to sleep in the attic of the main hospital building, or else in rooms only just vacated by the day nurses. Moreover, in order to reach their bedrooms, the nurses are obliged to quit the hospital building and incur the inconvenience and risk of frequent exposure to inclement weather; and the rent of the hired premises amounts to the substantial sum of $300 per year.

No more need be added to show that, apart from the introduction of the district nurse, the accommodation for the ordinary staff is utterly inadequate. The trustees have therefore decided to take the opportunity offered by the erection of a new home, at a cost of $25,000, to fulfil the original mission of the hospital, and provide rooms for at least 24 nurses, as well as the superintendent. The home is, of course, to be connected with the main building by a covered way.

THE FIRST DISTRICT NURSES.

Dr. Alfred Worcester, of Waltham, in recommending the inauguration of the district nursing system, said that he could never begin to speak about district or any other branch of nursing without going back in his own mind to the beginning of the nursing vocation. We have, he maintained, such a clear date for it. "The women who walked in the actual footsteps of the Master, who heard Him preach, who saw His infinitely tender relationship with the sick and suffering, after His crucifixion felt it to be their duty to be nurses. While the men preached, they thus practised what the Master taught, and these early Deaconesses in the Christian Church must, I think, have had as much to do with the spread of the Christian religion as the preachers who taught from the pulpit."

NURSING A VOCATION.

Dr. Worcester went on to express his opinion that the Deaconess orders which flourished in the early Christian ages had probably been supplanted by institutions solely devoted to the profession of nursing for the same reason that led to the separation of the medical from the clerical profession. "We should hardly," he continued, "want to go back to the time when the minister and the doctor were one and the same. Nursing is now the vocation of woman, instead of the avocation. The old Deaconess nurses felt that their most important work was to preach, and that nursing was a secondary duty. Now the world feels that nursing is quite enough of a profession for any woman to be primarily and wholly dedicated to."

DISTRICT TRAINING ESSENTIAL.

Insisting upon the absolute necessity of proper training for all engaged in nursing, Dr. Worcester argued in favour of nurses in every training school being afforded the chance of going out into service in district training. It was not fair, in his judgment, to nurses not to train them in "this distinct and important branch of the profession." He claimed that while the science of nursing could best be taught in hospital wards, the art of nursing could best be taught in the sick chamber, and concluded by affirming his conviction that if doctors could only find time to tell the difference in the scene of the birth of a child with no nurse; in the sickness of the mother or the father; in the death of the child or the aged; the difference between the situation where there is a nurse and where there is none; "every single man and woman belonging to the Christian churches would feel it to be a duty above every other to make sure that henceforth no child in the neighbourhood or the city of Boston should ever again be born without a loving welcome awaiting it; and that no man or woman, however much of a wreck, should ever hereafter be allowed to die unattended."

Several other speakers endorsed the view of Dr. Worcester, and the President of the Board of Trustees, Mr. Edward Haskell, in congratulating the graduates who had completed their course of study in the training school of the hospital, and handing them their certificates, addressed some well-chosen words of encouragement to them "to bring added honour to their worthy superintendent and matron" by the manner in which they exercised their calling.

### Where to Go.

THE WOODBURY GALLERY, 37 NEW BOND STREET.—Mr. Maurice Randall is now showing a most charming collection of marine pictures at this gallery. They are well worth a visit. They are well hung in the quiet little gallery.

### To Nurses.

**WE invite contributions from any of our readers, and shall be glad to pay for "Notes on News from the Nursing World," or for articles describing nursing experiences, or dealing with any nursing question from an original point of view. The minimum payment for contributions is 5s., but we welcome interesting contributions of a column, or a page, in length. It may be added that notices of appointments, entertainments, presentations, and deaths are not paid for, but that we are always glad to receive them. All rejected manuscripts are returned in due course, and all payments for manuscripts used are made as early as possible after the beginning of each quarter.**

### New Year and Christmas in the Hospitals.

By Our Own Commissioners.

(*Concluded from page* 191.)

King's College.

On Christmas Day the patients at King's College Hospital had a Christmas dinner of turkey and plum pudding, the wards being tastefully decorated by the medical staff and the nurses. But the general Christmas entertainment, which is always awaited with eagerness, took place on Boxing Day, when, as on former occasions, a number of members of the staff visited most of the wards, bent upon affording amusement. The programme consisted of a display of historical waxworks, songs by a nurses' glee party and by six pierrots, and recitations. An enthusiastic reception was given to the performers who entered with spirit into the undertaking, and seemed to enjoy it as much as the audience. On Saturday the children had a special entertainment to themselves, each of them receiving a share of the spoils of a Christmas-tree well furnished with toys. In consequence of the small-pox epidemic visitors were not admitted, but the proceedings were throughout admirably arranged.

Great Northern.

Christmas fare was the order of the day at the Great Northern Central Hospital, Holloway Road, the turkeys being subscribed for by the staff. Dinner was at twelve, and in the afternoon the patients' friends came to see them, and the members of the Ladies' Association presided over tea. A tree was also given by the association, and a band of carol singers visited each ward in turn and gave a selection of carols and songs.

Cancer Hospital.

There were plenty of presents this year for the patients at the Cancer Hospital, Fulham Road, and a full and varied programme of amusements was provided for Christmas week. On Christmas Day, carols were sung by the nurses, the ward doors being opened. The wards were all gay with flowers, art muslin, coloured lamp-shades and evergreens, each sister being responsible for her own ward. There was the usual Christmas dinner, for which no one seemed to be any the worse. The rule as to the non-admission of friends was relaxed by the committee for the one day, and entertainments were provided in an empty ward on the evenings during the week. Mr. Harvey not only gave a very interesting magic-lantern exhibition, with views of the Royal Family and the voyage of the *Ophir*, but also two character sketches: "Mr. and Mrs. Alphonso Brown's Silver Wedding Party" and "Songs in Different Languages." Madame Beresford recited "In the Children's Hospital," and one of the nurses sang a duet called "All's Well" with Mr. Rogers, nephew of the matron.

London Temperance Hospital.

After the Christmas morning services, at the London Temperance Hospital, turkeys, crackers, and smoking were in the programme for the day, and a band of gentlemen and ladies sang nursery rhymes and carols in the wards; this was a new feature and was greatly enjoyed. Then the patients had their tea-party, those who were well enough going down subsequently to a decorated and illuminated empty ward to witness an entertainment consisting of magic lantern and concert. On Monday evening the ward was again lighted with electric light and Chinese lanterns, festoons of evergreen being arranged, hanging from the ceiling, over a splendid Christmas-tree loaded with presents. There was a room full of guests, including the majority of the patients and nurses, and an excellent musical programme was gone through. Mr. Alderman Strong presided, and Canon Fleming said a few words on the duty of temperance, and wished everyone a Happy New Year. He also recited "The Lady Clare," and the humorous story of a wedding, called "The Third Time of Asking." Three quartettes were enthusiastically received; also some Irish songs, and "The Milkmaid." The performance ended with a "Cat Duet," consisting of "miows" from three ladies, with piano accompaniment. After a few words from the chairman, in which he congratulated the new matron on a most successful entertainment, the secretary climbed a ladder and distributed the presents from the tree. No one was forgotten, and the presents for those patients unable to leave the wards were taken to them afterwards by the nurses.

Chelsea Hospital for Women.

The annual entertainment for the patients at Chelsea Hospital for Women was given last Saturday afternoon, the nurses' concert having taken place the previous Saturday. The decorations on the third floor, where the beds were shifted so that the occupants could easily see the performers on the stage, were very good. Under the clock which faced the stairs the legend of "Good Luck, 1902. Welcome to All and Any!" appeared upon a red ground. Evergreens and pretty shades to the electric lights played an important part, and in the ward itself a good effect was made by hanging tiny celluloid balls and other Christmas-tree ornaments from the shades. On the second floor scarlet was the principal colour employed, whilst the corridor was outlined with fairy lamps which gleamed at regular intervals all up both sides. On the first floor pink predominated in the corridor, but each ward had been treated in a separate manner, and they were particularly interesting in the way the idea had been worked out. One room was dedicated to India. Here on the table was a capital imitation of a jungle, out of which came many wild beasts, including elephants, tigers, crocodiles; Hindu men and women with dusky skin stood about; and on the lockers and elsewhere sat dolls, male and female, attired in Eastern costume. The scarlet and gold lamp shades were quite in keeping with the brilliance of the Eastern colouring. On the opposite side was the Japanese room. Paper handkerchiefs with a Japanese border shaded the lights; pink almond blossom (which had been most cleverly copied by the originator of the scheme) branched out brilliantly here and there; and the group on the table consisted of an enormous Japanese boat across which were strung wee Japanese lamps, whilst Japanese groups stood at ease or manned the boat. The white sacred doves hovered over all. Above the door of another ward reminiscent of the Willow Pattern plate was a framed set-piece in which Japanese dolls of various sorts and sizes held up blue woollen letters wishing all "A Jappy New Year." The other wards were dedicated to nursery rhymes. In one Bopeep with her handkerchief to her eyes sat and sorrowed for her flock of white woolly sheep which had strayed to the edge of the table, and were hidden by diminutive trees and shrubs represented by ferns and foliage; in another Hansel and Gretel were nearing the sugar house in the woods from which the witch was emerging, the surrounding wood being redolent of the real violets strewn amongst the moss; while yet a third had demonstrated the story of Red Riding Hood, with a tiny doll discovered resting in the wood, whilst a wolf—who had much taxed the ingenuity of the nurse, because he could not be bought, and had to be manufactured out of a dog by the cutting of his ears and the addition of a red tongue—approached at a distance. This tableau had, however, to be removed early, owing to a serious case being brought in. Capital entertainments were given simultaneously on the first and third floors, and were much appreciated. The performers included Miss Nellie Ganthony, Miss Vera Beringer, Mr. Lionel Brough, and Miss Ethel Wood.

Metropolitan Hospital.

The Christmas festivities at the Metropolitan Hospital began at 5 A.M. on Christmas morning, when a procession of the nurses and sisters carrying coloured lamps and singing carols made their way from ward to ward. The effect in the early morning light was very pretty, and one of the patients quaintly remarked that "it was like a lot of angels." Father Christmas, accompanied by a very lively clown, distributed presents during the morning, and every patient received a parcel containing some clothing suitable to each, while each of the children had a Christmas stocking filled with toys. After a substantial tea, to which every patient was entitled to invite a friend, the entertainments began, and consisted of a concert given by various friends of the hospital, a play in which the resident doctors took part, and an exhibition of Mrs. Jarley's waxworks by the matron and sisters. All the patients had a thoroughly happy day, and one man said that "he wished he had known that 'orspitals were so nice at Christmas; he would have tried to be ill before." The children's Christmas-tree entertainment took place on January 2nd, and many friends of the hospital accepted the invitation of the committee to be present. The children's ward was prettily decorated, and a large tree, 13 ft. high, was laden with toys and presents of every description. After the tree was lighted up the presents were distributed, and the children were delighted with their various toys. Everyone admired the decorations of the wards and staircases, which looked quite brilliant with fairy lights and plants.

Hospital for Sick Children.

Every ward in the Children's Hospital, Great Ormond Street, was turned into a glade in fairyland on New Year's Eve. In one, the lights were blue, and the children wore blue jackets and blue ribbons in their hair; in another, the prevailing colour was red; and in yet another, it was yellow. There were strings of fairy lamps across the ceiling, and strings of beads glittered on the Christmas trees, which streamed with silver showers of rain. Each ward had its specially attractive feature; "Alice" had a new rocking elephant, "Alexandra" boasted a wonderful sugar church, with sugar trees outside and sugar bells in the tower, while "Helena" had its musical nurses, who sang and played such songs as the children like. One tiny mite, her hair and sleeves tied with pink ribbons, went to church all on her own account, for her nurse, going in search of her, found her quietly sitting on a bench in the chapel gazing at the white flowers with which this beautiful little sanctuary was decorated. At length, however, half-past four struck, and then someone began to light the tiny candles, and soon there was blaze of light and colour; then a ladder was brought, and the fruit began to come down from the tree, and was carried to the cots to be eagerly examined by the owners. A few of the children, especially the little ones, looked almost too ill to enjoy the fun; but others were very wide awake and happy, as indeed they might well be, for no pains were spared by doctors, nurses, or visitors to make the afternoon a happy one for the sick children.

East London Hospital for Children.

On Thursday last the inmates of the East London Hospital for Children at Shadwell had their annual treat. They had a splendid time. Sir T. Fowell Buxton gave two beautiful Christmas-trees, which were loaded with gifts sent by friends of the hospital, including the Princess of Wales and her children. Every child was remembered, and it was a great source of pleasure to the Staff that a very large proportion of the little invalids were well enough to be out of bed, though not all strong enough to sit up. These the nurses carried to the hall and laid on mattresses soft with surrounding pillows, so that they were able to enjoy not only the trees and the music, but also the cinematograph pictures.

Homœopathic Hospital.

New Year's Eve was the children's evening at the Homœopathic Hospital, and their ward was gay with trailing ivy, smilax, and flowers, while an enormous tree seemed to take up nearly the whole of one end. There were many visitors, and music and singing went on at intervals during the afternoon. The nurses themselves sang "I know a bank," and other songs were contributed by friends. The patients had already received their presents, these being distributed on Christmas Eve; and on Christmas Day, being visitors' day they had had their friends to see them, so New Year's Eve might be said to finish their round of festivities.

The Orthopædic Hospitals.

At the Royal Orthopædic Hospital, carols were sung on Christmas Day by the matron and nurses in the wards, with the help of the house surgeon. "The Londoners" have kindly promised to give an entertainment for the amusement of the patients on January 7th, at seven o'clock. At the National Orthopædic Hospital, a service was held in the morning by the chaplain, when carols were sung. After dinner, all the adult patients gathered in one ward and had fruit and crackers, and more carols were sung in the afternoon. There were games and singing in the children's wards. The Christmas-tree, when everyone in the house receives a gift, will be on January 11th.

The Hospital and Home for Incurable Children, Maida Vale, W.

The inmates of the Hospital and Home for Incurable Children, Maida Vale, spent a very bright and happy Christmas. They were remembered by many kind friends of the institution, but a new and wonderful joy was experienced by them this Christmas, when they received a gift of toys and of chocolate from the Queen. On the afternoon of Wednesday, January 15th, from 3 to 6 P.M., there is a Christmas entertainment at this home, and from 3 to 4 the children will sing carols.

East End Mothers' Home.

The Christmas festivities at the East End Mothers' Home were kept exclusively for the in-patients and their husbands. Through the kindness of many friends each patient received for herself and baby a parcel of useful clothes as well as a pretty Christmas card, the annual gift of the Countess of Harrowby. Those at home were not forgotten, each husband and eldest child receiving a small gift. Owing to the liberality of a friend the mothers enjoyed a good dinner, consisting of turkey—an unknown luxury to many—and custard puddings; the mothers who were able dined together, the table being prettily decorated with flowers and fairy lights. The husbands were invited to tea at the Home. Carol singing and a magic-lantern helped to make a pleasant evening for all.

Brentford Union Infirmary, Isleworth.

Christmas Day began early at Brentford Infirmary. The nurses had the necessary work to do and were anxious to put the finishing touches to the decorations. All the patients found on their pillow when they awoke a Christmas letter, and the children, in addition, the stocking they had hung up overnight well filled with gifts from their nurses and a toy from *Truth*. One little girl of five years had hoped Father Christmas would bring her a cradle to put dollies in, and great was her delight to discover one on her bed in the morning. She went about all day showing it. A short service was held by the chaplain in the morning at which most of the convalescent patients were present. Though the styles of decoration in the wards were very different, all reflected credit on the sisters, nurses, and probationers, who had worked so willingly in order to give pleasure to the patients. In front of one ward was an imitation pond formed of glass on which appeared to float cleverly made white wool ducks. Rising from a centre table in another ward was a lighthouse, made by the night nurse. Overhead from a festoon were the devices, "Success to our Doctors," "Health and Happiness to our Matron." The dinner of roast beef and plum pudding was served in the wards, the tables being prettily decorated with flowers and crackers. After dinner the convalescents were allowed to visit the other wards, to admire the decorations and chat with those not well enough to get up. The medical superintendent kindly arranged two concerts in the afternoon. After tea the matron and sisters, nurses, and probationers sang carols in the wards in turn.

St. Mary's Hospital for Sick Children, Plaistow,

The annual entertainment for the children in St. Mary's Hospital for Sick Children at Plaistow was held on Friday. The wards and corridors were tastefully decorated for the occasion by the resident doctors, the matron, the sisters, and the nursing staff. Christmas trees (the gift of Mr. T. F. Buxton, of Ware) which were laden with toys and games, presented by many kind friends in all parts of the country, gladdened the hearts of the youngsters, who thoroughly appreciated the good things provided for them. A small band of the East London Engineers enlivened the proceedings, whilst a Punch and Judy show was not the least attractive of the several amusements. Many friends interested in the work of the institution were present.

The Station Hospital, Portsmouth.

On Christmas Day all the convalescent patients at the Station Hospital, Portsmouth, to the number of about 80, sat down to roast beef and plum pudding for dinner in prettily decorated wards, and on Boxing Day a capital tea was arranged by the sisters on two long tables in an empty ward, decorated for the occasion with scarlet cloth and evergreens. After tea, the tables being removed, the room was arranged for a smoking concert. Sergeant Robertson, of the Gordon Highlanders, Miss Colman, and the Rev. F. Tremble kindly contributed several charming items to the programme, and some of the patients who had the gift of song also lent their aid.

Royal Hants County Hospital, Winchester.

Christmas was well observed at the Royal Hants County Hospital. All the wards were extremely prettily decorated by the sisters and nurses. Christmas Day began with services in the chapel, and the patients dined at noon on turkeys, plum puddings, jelly, etc. The servants had their dinner afterwards. During dinner they had their presents in a snowball presided over by Father Christmas. Wednesday being visiting day, the patients were allowed to have one friend each to tea. Service in the chapel was followed by the singing of carols in the wards, and later in the evening all the staff dined together. There were tea parties during the week in the various wards, a very nice concert in Bartlett ward on Friday, and on Monday a concert in Victoria, followed on Tuesday by a Christmas-tree, very prettily decorated, in Heathcote, which many members of the committee and friends attended. The matron's Christmas present from the sisters and nurses was a dressing-bag, and she received a silver toast-rack from the servants.

South Devon and East Cornwall Hospital.

Christmas at the South Devon Hospital has been a happy time for all. The festivities were kept up for quite 10 days. Christmas Day itself was spent quietly. The patients had turkey and plum-pudding; after tea the nurses sang carols in the different wards. On Boxing night the matron, doctors, chaplain, and a large staff of nurses had their Christmas dinner at 7 p.m. Sister Hainslin, formerly of the South Devon Hospital, and who has lately returned from the war in South Africa, was also present. There has been tea in each ward, to which all the nurses, a few visitors, and as many patients as could go were invited. Afterwards there was singing, and "living pictures" were given by the doctors, sisters, and nurses. On the 30th all enjoyed a splendid entertainment and refreshments given by the house surgeons. The children were amused on Christmas morning by one of the nurses dressing as Father Christmas, and giving them each a toy. Thursday was finished with a tea and Christmas-tree in the children's ward.

Bridgnorth Infirmary.

The festivities at the Bridgnorth Infirmary on Christmas Day commenced early in the morning with a visit from "Santa Claus" carrying a Christmas-tree laden with presents for each patient. These were distributed by the nurses, after which a Christmas hymn was sung. There was the usual Christmas dinner. In the afternoon a tea was given in the wards to the patients and their friends. Subsequently much amusement was afforded by a phonograph, which was described by one old woman as a "talking machine." The fun of the evening also included a Fish Pond, each patient fishing in turn, and great was the excitement when a "fish" was hooked. The parcels were folded up to represent various fish.

West Cornwall Miners' and Women's Hospital.

On Christmas Eve the West Cornwall Miners' and Women's Hospitals at Redruth presented a gay and festive appearance, the decorations of scarlet berry and variegated holly, evergreens, and Chinese lanterns, being tasteful and effective. Attractions at the "Nurses' At Home" included a Christmas tree for the inmates of the children's ward, with toys and substantial and useful presents for grown-ups and the little ones. Contributions for these gifts had been received from members of committee and medical staff, and collected by the nursing staff from outside friends. As usual geese and turkeys for the Christmas dinner were received from Mrs. Basset of Tehidy, President of the Women's Hospital; other gifts in kind were sent in by various friends. Christmas Day fare, including plum pudding, dessert, and Christmas cake, was thoroughly enjoyed. The Redruth Town Band played some Christmas music, and on the following Monday there was an excellent concert of both vocal and instrumental music.

Cuddington Hospital, Epsom.

On December 27th, and again on January 3rd, a quiet dance was given to the staff of the Cuddington Hospital, through the kindness and generosity of the committee. It was held in the new block, which has not yet been occupied, and was given on two nights in order to allow the nurses to attend in detachments. The staff were in uniform and the dance was well attended by outside friends.

Hertford British Hospital, Paris.

Not the least important among the many charitable entertainments with which Christmas Day was celebrated was that given at the Hertford British Hospital, Levallois-Perret, Paris. The inmates were regaled with a succulent repast, consisting principally of the traditional roast beef and plum pudding, followed by a suitable dessert. The whole of the staff, medical and nursing, dined together, Miss Neech, the matron, paying particular attention to the welfare of all present This enjoyable dinner was followed by a sacred concert, specially organised for the occasion by Mr. Reginald Gesling, the secretary. The singers, members of the choir of the American Church, Rue de Berri, included Mmes. Mathieu, Donnante, and Bourgeois, all of the Opéra, aided by Mlle. Bourgeois and the two sons of Mme. Mathieu. The proceedings terminated with warm votes of thanks to the singers and to the organisers of this enjoyable function.

### Appointments.

CHESTERFIELD AND NORTH DERBYSHIRE HOSPITAL.—Miss E. Reid Dobie has been appointed matron. She was trained at the Western Infirmary, Glasgow, for three years, and was then appointed charge nurse, afterwards going as night superintendent to the Royal Infirmary, Bradford, where she also served as housekeeper for a year. During the last three years Miss Dobie has been assistant matron of the General Hospital, Birmingham.

COUNTY ASYLUM, RAINHILL, LIVERPOOL.—Miss Annie Louise Williams has been appointed head nurse. She was trained at the Royal Infirmary, Glasgow.

DARENTH FEVER HOSPITAL, SOUTH SHIELDS.—Miss Helen A. Munro has been appointed charge nurse. She was trained at Knightswood Hospital, Glasgow, and has since been assistant nurse in the same institution.

EAST PILTON HOSPITAL, EDINBURGH.—Miss Katharine Lunn has been appointed sister of the scarlet fever wards. She was trained at East Pilton Hospital and at Salop Infirmary.

EAST LONDON NURSING SOCIETY. — Miss Lillian Mary Davidson has been appointed nurse for the district of All Saints, Buxton Street, E. She was trained at the Canterbury Workhouse Infirmary, and has since held the posts of staff nurse at Chelsea Infirmary, St. Anne's Home, Herne Bay, and the Willesden Temporary Infirmary. She has also done private nursing.

HOSPITAL FOR CONSUMPTION, VENTNOR.—Miss Ethel Lloyd has been appointed assistant matron. She was trained at the General Hospital, Birmingham, for three years, at the end of which time she was promoted to be sister of one of the female medical wards, which appointment she has held for the last three and a half years.

INFECTIOUS DISEASES HOSPITAL, BLACKPOOL. — Miss Florence Brown has been appointed matron. She was trained at the Royal Albert Hospital, Devonport, and has since been charge nurse at Northampton General Infirmary, charge nurse at Grafton Street Hospital, Liverpool, and assistant matron at the City Hospital, Dingle, Liverpool.

LAMBETH INFIRMARY.—Miss Marian Longroos has been appointed charge nurse. She was trained at University College Hospital, London, for three years, and was afterwards, for a year, on the private nursing staff. She has since been staff nurse at Brompton Chest Hospital.

LONDON SCHOOL BOARD.—Miss Florence M. Boyce has been appointed lady lecturer on nursing to the School Board for London. She was trained at the North London Hospital for Consumption and Diseases of the Chest, and also for three years at the Mile End Infirmary. She has since been attached to the All Saints' Nursing Institution, and engaged in private nursing.

NEWCASTLE-ON-TYNE UNION HOSPITAL. — Miss Jessie Jayne and Miss B. C. Dean have been appointed charge nurses. Miss Jayne was trained at St. Bartholomew's Hospital, London, and Miss Dean at Chorlton Union Infirmary, Manchester.

POPLAR UNION INFIRMARY.—Miss Elizabeth W. Lees has been appointed nurse. She was trained at Lambeth Infirmary, and has since been nurse in that institution, and also at the Poplar Union Infirmary, to which she now returns.

ROYAL HANTS COUNTY HOSPITAL, WINCHESTER.—Miss C. M. Crawford has been appointed sister of the male medical ward. She was trained at Leeds Infirmary, and has since been sister at Grimsby Hospital, night sister at Bolton Infirmary, and sister at Wirral Children's Hospital, Birkenhead.

SOUTHAMPTON WORKHOUSE INFIRMARY. — Miss Jessie Fraser Ballantyne has been appointed matron. She was trained at the Cottage Hospital, High Barnet, and Guy's Hospital, London. She has since been sister, and lately assistant matron at Lewisham Infirmary.

THE SANATORIUM, MORECAMBE, LANCASHIRE. — Miss Valetta Shout has been appointed matron. She was trained at the Alexandra Hospital for Children, London, and Addenbrooke's Hospital, Cambridge, and has since been staff nurse at the Sanatorium, Hull, charge nurse at the City Hospital, South Grafton Street, Liverpool, and matron at a convalescent home at Skegness.

### Presentations.

BLACKPOOL SANATORIUM.—Miss Cain, matron of Blackpool Sanatorium, who leaves England this week to take up her new duties as matron of the City Hospital, Capetown, has been presented, by her probationers, with a set of silver-backed hair brushes, combs, and clothes brushes. Nurses Serase and Blair, who accompany her, have also received appointments in the same hospital.

COTTAGE HOSPITAL, CHIPPENHAM.—Miss Maud Melton, who has just completed her training as probationer at the Cottage Hospital, Chippenham, has been presented, on leaving, with a handsome carriage clock, bearing a suitable inscription, by the ladies' committee, and with a suitably-fitted writing-case by the committee of management.

LLOYD HOSPITAL, BRIDLINGTON.—Miss A. Maud Jones, on her retirement from the post of matron at the Lloyd Hospital, Bridlington, received a very handsome silver tea set given by the general committee and the medical staff. The ladies' committee presented a silver kettle and stand with spirit lamp attached, and silver teaspoons, each article bearing the monogram of Miss Jones. The nursing staff presented a very beautiful silver-mounted oak tea tray as a mark of their good wishes and esteem.

UNION INFIRMARY, SUNDERLAND.—On Christmas morning, at the Sunderland Union Infirmary, Miss Pruett, superintendent of nurses, was presented with a handsomely-chased silver sugar-basin and cream-jug, by the nursing staff, as a token of the esteem in which she is held.

### Death in Our Ranks.

WE regret to announce the death, on Christmas Eve, of Mrs. Allen, who for upwards of 20 years was one of the nurses at the Royal Isle of Wight Infirmary, Ryde. She retired in 1898, but, through the kindness of the committee, by whom she was much esteemed, remained at the Convalescent Home until January, 1901, when she removed to Oxford, where she hoped to qualify for one of the almshouses, or pensions, in the gift of the University.

### Novelties for Nurses.

BY OUR SHOPPING CORRESPONDENT.

### IDEAL BLANKETS.

VIYELLA flannels have become almost household necessities, and there is hardly anyone who does not possess the excellent material in some form. The latest novelty that has been brought to my notice is the Viyella blanket. This blanket has just the same soft finish, and beautifully even firm consistency in common with all Viyella productions. It is an ideal blanket, because it is light and warm, and non-irritating to the skin. In the sick room it should certainly supersede ordinary blankets, which, however desirable for an invalid, are frequently discarded owing to the irritation they produce. In private hospitals and nursing homes Viyella blankets should be universally adopted. They do not shrink, and any draper will supply them.

### Everybody's Opinion.

"HOSPITAL GRATITUDE."

"Nurse B. B." writes: We hear a good deal about hospital gratitude. I remember when I was a probationer in one of our large London hospitals, an old man, about 80, was admitted from Whitechapel into one of the medical wards. He was a typical old "Grandpa," quite bald, with one front tooth, and he used to make a striking picture sitting up in his scarlet jacket, and a cap to match; but he was of an unhappy, discontented disposition, and was always complaining and finding fault with one and all the nurses. And one day, when he was more aggrieved than ever, his fellow sufferers remonstrated with him for his ingratitude to those who waited on him, and he replied. "Well, what have I to be grateful for? I tells you, them young persons is paid to wait upon I, and I means to get my money's worth!"

CHRISTMAS FARE AT A LYING-IN HOSPITAL.

"E. A. B." writes: I have been exceedingly surprised at reading the account of Christmas Day in the City of London Lying-in Hospital in which the writer describes a patient as entering the hospital at 11.30, her baby being born at 12.15, and at 12.30 as picking the wing of a chicken and being allowed to helpings of plum-pudding! I am a midwife (Q.C.H. and L.O.S.) of many years' experience and cannot but think that your commissioner is in error. Why, 15 minutes after the birth of the child the placenta would hardly be expelled, and my teaching was always to wait 20 minutes if all was going on well before taking it, and certainly the mother could not have been put comfortable. Then one is taught that a light meal, egg and milk, or tea and toast, or warm milk is all that should be given after natural labour and the patient be then encouraged to rest and sleep if she can. I consider that such a statement, even if correct (which I doubt), is most dangerous, as an ignorant person seeing it would naturally think in the face of it that it did not matter what was given after a confinement. Of course, one knows that a much more generous diet is allowed now than, say, 50 years ago, but I for one should be very sorry to imperil a patient's life by allowing her chicken and plum-pudding before her baby was half an hour old!

INFLAMMABLE GARMENTS.

"E. L. M." writes: Reading the other day of an inquest held on a child burned through her flannelette garments catching fire I can only wonder that cases are not happening every day, because of the most inflammable nature of the material. I am also speaking from experience, for some winters ago I bought a very pretty and warm flannelette dressing-gown. Happening to pass too near a gas stove, which was on the ground, I saw, to my horror, in a long glass, that I was all ablaze. In a second the flames seemed to fly up from the bottom of the gown to the top; also the sleeves caught fire directly. With presence of mind I was able to put myself out, and what was my surprise to find the gown did not instantly drop to pieces; in fact, except for the smell of burning, there was nothing to denote I had been on fire, for only the surface or fluff seemed to have really been burnt away. Flannelette being so cheap and also warm is in the reach of every poor woman's purse, and is much used for the children for undergarments. But as in the same way we receive a label of poison with any dangerous drug, so I do think we ought to have a printed notice of danger with such a highly inflammable material, and then we should at least be more careful, and there would be no excuse that we went too near the gas or fire and did not know. Will you kindly tell me who is responsible for a danger like this, and to whom one goes to have it rectified?

[No one is responsible for the fact that some materials are more inflammable than others. The utmost that can be done is for district nurses and others who work amongst the poor to impress upon all whom they come across that it is dangerous for flannelette garments to be brought into contact with fire or gas. If needed, a scrap of flannelette would soon supply a practical object lesson.—Ed. The Hospital.]

### For Reading to the Sick.

THE PEACE OF GOD.

Would'st thou possess this peace? be still, be low!
Peace with the pure abides;
Yea, all the humble, all the gentle, know
The shelter where she hides;
Rooted in patience, her fair buds to flowers shall grow.

Happy are they that learn, in Thee,
Though patient suffering teach
The secret of enduring strength,
And praise too deep for speech—
Peace that no pressure from without,
No strife within, can reach.

*Anna L. Waring.*

Grant to me above all things that can be desired, to rest in Thee, and in Thee to have my heart at peace. Thou art the true peace of the heart, Thou its only rest; out of Thee all things are hard and restless. In this very peace, that is, in Thee, the One Chiefest Eternal Good, I will sleep and rest. Amen.—*Thomas à Kempis.*

Peace is the tranquility of order—calm in the presence of God, calm in the felt superiority over all assaults of the enemy, calm in full submission to God's merciful ordering; and, when tie after tie is snapped, and the golden threads which bind us down to earth are cut away one by one, in the great severance of death itself, still—Peace. Is anxiety sent from God? Has He not said, "Take no anxious thought," "casting (down) all your care upon Him, for He careth for you." It is we who drop the hand of God and try to walk alone. It is we who cast ourselves out of the ship, and try to walk on the water of trouble to go to Jesus, and fail because of our want of faith. "He that believeth shall not make haste." Yet, sitting still, patient and expectant, we have learnt to love, and from Love has streamed out the radiance of Joy, and with Love and Joy comes Peace. And, stopping to ask ourselves what is Peace, we say it is the abiding sense of God's presence and might within us, against which the troubles of life beat without power and break without overwhelming.

*Canon Newbolt.*

"These things write we unto you, that your joy may be full." What is fulness of joy but *peace*? Joy is tumultuous only when it is not full; but peace is the privilege of those who are "filled with the knowledge of the glory of the Lord, as the waters cover the sea." "Thou wilt keep him in perfect peace, whose mind is stayed on Thee, because he trusteth in thee." It is peace, springing from trust and innocence, and then overflowing in love towards all around him.—*T. H. N.*

Just to let thy Father do
What He will;
Just to know that He is true,
And be still;
Just to trust Him, this is all!
Then the day will surely be
Peaceful, whatsoe'er befall,
Bright and blessed, calm and free.

*F. R. Havergal.*

### Notes and Queries.

**The Editor is always willing to answer in this column, without any fee, all reasonable questions, as soon as possible.**
**But the following rules must be carefully observed:—**
**1. Every communication must be accompanied by the name and address of the writer.**
**2. The question must always bear upon nursing, directly or indirectly.**
**If an answer is required by letter a fee of half-a-crown must be enclosed with the note containing the inquiry.**

*Address.*

The *Asylum News* is published at 8 King Street, Richmond, Surrey, and not at Lancaster, as stated in answer 124.

*Leeches.*

(134) Please tell me (1) What is meant by the "application of leeches internally"—are they so used in the present day? (2) What becomes of stitches which are put in internal organs after operation? Do they come away when the wound is healed, and, if so, are they not free in the peritoneum?—*Ignorama.*

(1) There are certain places which, although they may be regarded as "internal," are still accessible to treatment by leeches. Such a spot, for example, is the cervix of the uterus. (2) Some kinds of sutures, as, for example, those made of catgut, are absorbed. Others become surrounded by an organised effusion and remain permanently, just as bullets often do, without exciting irritation. But they do not float about loose in the peritoneal cavity.

*The L.O.S.*

(135) In order to go for the L.O.S., is it necessary to have 20 vaginal examinations, or would not palpation and auscultation do as well? An answer will greatly oblige.—*U.U.K.*

Whatever the regulations of the L.O.S. are you must abide with them. A vaginal examination is an absolutely distinct and separate thing from palpation and auscultation, and 20 years spent in practising the latter would not give the student the slightest experience in the former. The demand for a given number of a particular form of examination is not to test knowledge but experience—experience of that particular kind.

*Medicines.*

(136) Will you kindly tell me the correct time to give (*here the printer fails to decypher Chloe's hieroglyphics, but we believe they are meant for "t. d." or "ter dice," or "three times a day."* The sisters under whom I trained gave them at 8 A.M., when they came on duty; again about 2 and 6. I am now told that 10 A.M. is the correct time, is that so? 2. What is Clover's crutch?—*Chloe.*

1. Chloe should avoid using abbreviations, and if she must use them, should write more carefully. When patients are aroused and washed, and have their breakfasts given early in the morning, they can take their medicine early, but if the day starts late so must the medicine. Breakfast sets the patient's clock. You would hardly begin giving a "three times a day" medicine on an empty stomach unless specially directed to do so. 2. A "Clover's crutch" is an apparatus, by aid of which the legs may be held apart in the "lithotomy position."

*Lavatory Utensils.*

(137) Will you kindly tell me what is the best and most approved arrangement for keeping the utensils in lavatories? Are shelves of wood the correct thing or is there any enamelled iron arrangement where it is impossible to have an outside cupboard opening in the wall?

If shelves are properly arranged so as to leave a space behind between them and the wall, and are made of hard white wood, and are well scrubbed with strong soap and water, and perhaps with a little sand, they will do very well. Enamelled iron things are very apt to crack—both themselves and the utensils—and painted things soon get rubbed and rusty. The wall behind the shelves should be done in glazed tiles set very close together, or else in a hard cement frequently painted in oil paint.

*Fibroid Disease of the Lung.*

(138) Will you kindly explain the meaning of the term "Fibroid Disease of the Lung?" Is it actually tubercular disease, or is it a state of the tissue previous to the deposit of tubercle?—*Inquirer.*

Fibroid disease of the lung or elsewhere is a degenerative process by which the tissues become changed into a fibroid material, like scar tissue, which tends to contract. It is not a tuberculous process at all, but it occurs around tuberculous deposits, and it is largely by this means that these deposits are isolated. So far as it occurs around tuberculous deposits it is to be regarded as a curative process.

*Abroad.*

(139) Can you tell me how I can get abroad, anywhere? I have had 12 months' training in a fever hospital and 14 months' in a general infirmary.—*M. F. C.*

You must have a three years' certificate in general training to be eligible for appointments through any good nursing association. Your only chance is to advertise for a private patient.

*Restless.*

(140) Will you kindly tell me the correct definition of "restless?" Does it mean a patient getting out of bed and moving about the room, or only moving about in bed?—*M. H.*

Restless means unable to rest either in or out of bed.

*Gynæcological Nursing.*

(141) 1. I should be obliged if you will tell me the numbers of THE HOSPITAL *Nursing Section* in which the lectures on Gynæcological nursing appeared, and (2) also, if there is any book published on the same subject? 3. Can you tell me where I can obtain information as to Queen Alexandra's Imperial Nursing Service?—*K. D. M.*

1. Lectures to Midwifery Nurses appeared on January 12th and 26th and in February 9th, 1901. 2. "Gynæcological Nursing," by G. A. Hawkins-Ambler, F.R.C.S., price 1s. (Scientific Press.) 3. Full details appeared in the *Nursing Section* of THE HOSPITAL on October 5th.

*Comparison.*

(142) Will you kindly tell me if a three-year certificate from an infirmary such as East Dulwich is equivalent to the certificate of a general hospital?—*G. M. H.*

If you mean Southwark Infirmary, East Dulwich Grove, S.E., the three years' certificate is recognised as full nursing qualification for the post of superintendent nurse by the Local Government Board; and is equal, in that respect, to one given by a general hospital recognised by that body as a training school for nurses.

*Male Nurse.*

(143) Will you kindly tell me how a young man of twenty can train as a male nurse? He has been an orderly in a military hospital for six months.—*C. J.*

Apply to the matron, the National Hospital for the Paralysed and Epileptic, Queen's Square, Bloomsbury, W.C. Failing that he may obtain the certificate of the Medico-Psychological Society by entering for three years an asylum which prepares for the examination of that body.

*Older Nurses.*

(144) Please let me know if there is a society for employing older nurses.—*Old Nurse.*

There is one for the older members of the Royal British Nurses' Association, 10 Orchard Street, W.

*Certificate of Baptism.*

(145) Will you kindly tell me if a certificate of baptism is required at all hospitals, and if so, is it required at the time of application, or at the end of the trial months?—*J. H.*

If the matron to whom you write in the first instance thinks you a suitable candidate for training she will send you a form of application and full directions how to proceed and what certificates are needed.

*Rome.*

(146) Will you kindly let me know the addresses of English nurses' institutions in Rome.—*Nurse C.*

The Anglo-American Nursing Home, Via Nomentana, Rome.

*Nursing Homes.*

(147) Will you tell me if there are any nursing homes in London which employ untrained nurses, or if there is any means by which nurse attendants can obtain a post other than by advertisement. *A. M. H.*

Only private nursing homes or registry offices would secure posts for untrained attendants.

*Light Employment.*

(148) Could you tell me how I could help a girl, partly crippled with rheumatism, to obtain light employment as sewing maid or something of the kind. She has been ordered to try a warmer climate—South Africa or the Canaries. To whom should she apply?—*Canaries.*

This will most probably have to be found privately. If the case is capable of improvement the patient would be eligible for treatment at some of the mineral water baths in England. There is no fund for sending such cases abroad.

**Standard Books of Reference.**

"The Nursing Profession: How and Where to Train." 2s. net; post free 2s. 4d.
"Burdett's Official Nursing Directory." 3s. net; post free, 3s. 4d.
"Burdett's Hospitals and Charities." 5s.
"The Nurses' Dictionary of Medical Terms." 2s.
"Burdett's Series of Nursing Text-Books." 1s. each.
"A Handbook for Nurses." (Illustrated). 5s.
"Nursing: Its Theory and Practice." New Edition. 3s. 6d.
"Helps in Sickness and to Health." Fifteenth Thousand. 5s.
"The Physiological Feeding of Infants." 1s.
"The Physiological Nursery Chart." 1s.; post free, 1s. 3d.
"Hospital Expenditure: The Commissariat." 2s. 6d.

All these are published by the SCIENTIFIC PRESS, Ltd., and may be obtained through any bookseller or direct from the publishers, 28 and 29 Southampton Street, London, W.C.

### Travel Notes.

By Our Travelling Correspondent.

LXXXIX.—AGAIN IN ROME.

In accordance with many kindly-expressed wishes, I purpose continuing our rambles in Rome during these dark winter days, and thus, in gloom and fog, prepare ourselves for future happy hours in the Eternal City.

The Quirinal.

This palace is now associated with the Royal Family of Italy, but in past times it was the Pope's home, and that rough, but upright, monarch Victor Emmanuel felt many qualms of conscience about inhabiting the Papal home, and would never occupy the actual rooms of his Holiness. In itself the Palazzo is not especially interesting; it lies to the right of the Piazza Monte Cavallo, with the beautiful statues of Castor and Pollux reining in their horses. There is much controversy as to the history of these splendid figures, bearing the names of Praxiteles and Phidias on their bases. Whatever their origin they are works of magnificence; what energy, what action, and what a knowledge of anatomy are displayed in them! The romantic interest of the Quirinal is centred in the history of the two Popes, Pius VII. and Pius IX., both of whom were compelled to fly from their beloved home—the latter never to return. Pius VII. was forced by Napoleon to abdicate his temporal sovereignty and to retire into exile. Cardinal Wiseman gives an interesting account of his hurried departure at that tyrant's command in 1809. "Whirled away through the heat and dust of an Italian summer day, without an attendant, without linen, without his spectacles—fevered and wearied, he never for a moment lost his serenity. . . . ." Six years later, when Napoleon was a prisoner at St. Helena, Pius VII. returned to the Holy City.

The Flight of Pio Nono.

The amiable and virtuous but weak Pope Pius IX. loved the Quirinal; it was there that he showed himself in the short time of his remarkable popularity standing on the balcony of the room that Queen Margherita used as a ball room. Mrs. Minto Elliot speaks with enthusiasm of the personal appearance of the Pope and of his lovable disposition.

"Some men, like women, are born beautiful. There is no doubt about it. You may not like the style, you may prefer blonde, you may admire black, but no one can dispute the beauty. Of these Pius is one. To his last day that precious gift continued to develop itself in all the changing phases of age. And there was more than beauty—a charm that Italians call *poesia*, a certain natural grace and refinement which under all circumstances never failed."—*Minto Elliot.*

In his youth, before entering the Church, and when preparing for the army, he was a great frequenter of the cafés, and it is supposed that in one of these he met Antonelli, then a young abbé. Thus began a close connection of upwards of thirty years, which resulted in disaster to his Holiness.

At the age of eighteen he showed symptoms of epilepsy, which, preventing a military life, was the cause of deep sorrow to him. Prayer was offered for and by him in the church of S. Maria degli Angeli, and it is certain that from that time he never had another fit. It was popularly supposed that it was in gratitude for this mercy that he entered the priesthood.

In 1848 his short period of glorious ascendency and remarkable popularity came to an end.

"Here" (speaking of the Quirinal) "he received all the ovations which an excitable and enthusiastic people could offer, sometimes called out in wind and rain, sometimes in the glare of fervent heat, late in the night, or in the first morning hours, to gratify them with his presence and to dispense his blessings.

"Two short years saw many thrilling scenes of love and devotion, many gorgeous pageants, when the temporal and spiritual power held by the Pope rendered him more than mortal in the eyes of men! But the dark days came apace, the cord was too tightly drawn, it needs must crack. Blameless of life, gracious, condescending, facile in a kind of familiar eloquence which touched all hearts, he was no statesman, nor possessed of the intrinsic qualities attributed to him, neither saint nor martyr. . . . He was incapable of commanding the position in which he found himself. Important reforms were granted too suddenly—what should have taken years was given in months."

—*Minto Elliot.*

These thoughtful words give an excellent idea of the causes which led to the downfall of the Papal temporal power. In 1848, after the murder of his minister Rossi, a popular tumult arose; the Quirinal was attacked and the gentle but incompetent Pope fled into the kingdom of Naples. His flight was managed by the Duc d'Harcourt, Ambassador of France, and Countess Spaur. This flight was a great mistake and political error. In 1871 it became the residence of King Victor Emmanuel, and the Pope now inhabits the Vatican in retirement and represents himself to the faithful as a prisoner and martyr.

Il Re Galantuomo, Victor Emmanuel.

This stalwart soldier, more fitted for the battlefield than the throne room, was ever a faithful son of the Church, despite his enforced antagonism to the Pope. I am sure that the "Blacks" of Rome would bitterly deny this statement, but it is true nevertheless. Circumstances forced him into this antagonism, and patriotism kept him there. His position called for great courage. The sacraments of the Church were denied him by the regular clergy, and he was without the pale. Mrs. Elliot seems doubtful whether his name ever actually appeared in any document of excommunication, but whether it did or not the result was practically the same. During a severe illness at San Rossore, absolution was refused him, and the great doors of Pisa Cathedral were closed against him. At Turin, when the weather was so hot that the cathedral doors were left open, the poor King would stand before them, and bow himself at the elevation of the Host that he was not permitted to witness nearer.

Victor Emmanuel was a strange and complex charactere devoted to his wife, he yet pained her by connections littl to his credit. Notably thus with Rosina, a waitress at a café, whom he created Countess of Mirafiore; this coarse and vulgar woman caused much misery and dissension in the Royal Family. She bore him several children, and eventually became his morganatic wife. She lived in a villa outside the Porta Pia, and died of cancer in 1878—the same year which also witnessed the deaths of Pio Nono and Victor Emmanuel.

The King died of rapid pneumonia, and there were many painful scenes before the dying man eventually received absolution and extreme unction, which were refused him unless he would sign a paper of restitution and submission to the Church. The kind heart of Pio Nono at length forced him into a different attitude. "The wretches," he said; "they want that poor man to die a heathen!" How it was managed is still a mystery, but absolution was given and the aged Pope sent him his pardon and blessing.

The Gardens of the Quirinal.

These now are seldom shown, but if you have an opportunity, through influence, to see them, they are well worth visiting, chiefly from the very beautiful views to be obtained from them.

The Palazzo Rospigliosi.

Close to the Quirinal is this palace, which is no longer open to the public; but in the Casino at the end of the garden are some gems of art, above all Guido's "Dance of the Hours" Mendelssohn makes rather a scathing remark to would-be critics in one of his letters home. "Guido's Aurora is the very type of haste and impetus; for surely no man ever imagined such hurry and tumult, such sounding and clashing. Painters maintain that it is lighted from two sides—they have my full permission to light theirs from three, if it will improve them, but the difference lies elsewhere!" Eaton says it is Guido's noblest work and embodied poetry, and truly one can pay many visits to it without weariness.

## Figures and Tables

**Fig. 18. f1:**
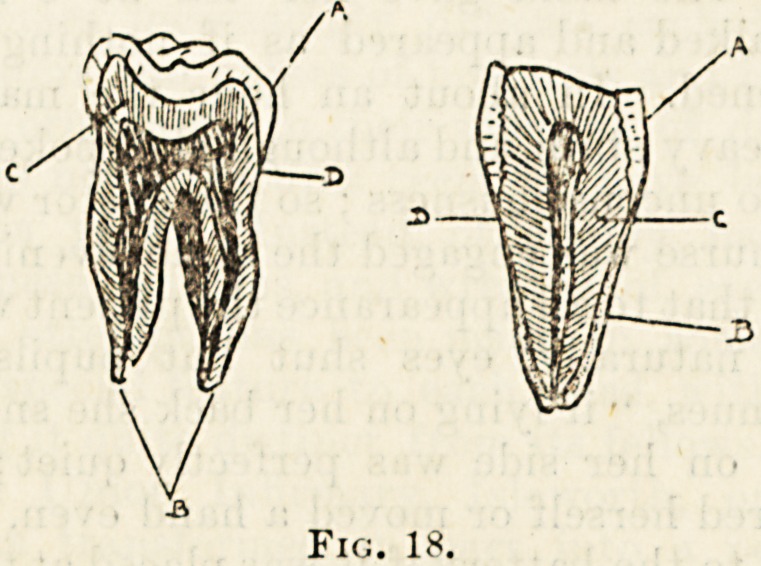


**Fig. 19. f2:**